# Racial and Geographic Differences in Breastfeeding — United States, 2011–2015

**DOI:** 10.15585/mmwr.mm6627a3

**Published:** 2017-07-14

**Authors:** Erica H. Anstey, Jian Chen, Laurie D. Elam-Evans, Cria G. Perrine

**Affiliations:** ^1^Division of Nutrition, Physical Activity and Obesity, National Center for Chronic Disease Prevention and Health Promotion, CDC; ^2^Immunization Services Division, National Center for Immunization and Respiratory Diseases, CDC.

Breastfeeding provides numerous health benefits for infants and mothers alike. The American Academy of Pediatrics recommends exclusive breastfeeding for approximately the first 6 months of life and continued breastfeeding with complementary foods through at least the first year (*1*). National estimates indicate substantial differences between non-Hispanic black (black) and non-Hispanic white (white) infants across breastfeeding indicators in the United States (*2*). CDC analyzed 2011–2015 National Immunization Survey (NIS) data for children born during 2010–2013 to describe breastfeeding initiation, exclusivity through 6 months and duration at 12 months among black and white infants. Among the 34 states (including the District of Columbia [DC]) with sufficient sample size (≥50 per group), initiation rates were significantly (p<0.05) lower among black infants than white infants in 23 states; in 14 of these states (primarily in the South and Midwest), the difference was at least 15 percentage points. A significant difference of at least 10 percentage points was identified in exclusive breastfeeding through 6 months in 12 states and in breastfeeding at 12 months in 22 states. Despite overall increases in breastfeeding rates for black and white infants over the last decade, racial disparities persist. Interventions specifically addressing barriers to breastfeeding for black women are needed.

NIS is a national ongoing, random-digit–dialed cellular and landline telephone survey conducted among households with children aged 19–35 months (*3*). The survey primarily is intended to estimate vaccination coverage rates for U.S. children. Questions on breastfeeding were added to the survey in 2001 and have since been used for national breastfeeding surveillance.

Because children are aged 19–35 months at the time of the NIS interview, each cross-sectional survey includes children born in 3 different calendar years. To increase sample size and allow for representative state-level analyses stratified by race, a cohort of children born during 2010–2013 was created by combining data from the 2011–2015 surveys. The Council of American Survey and Research Organizations response rates for the landline sample of NIS years 2011–2015 ranged from 59.2% to 76.1%. Response rates for the cellular telephone sample of NIS years 2011–2015 ranged from 25.2% to 33.5%. The child’s breastfeeding history and race/ethnicity, and the mother’s age, education, household percent of poverty level, and participation in the Supplemental Nutrition Program for Women, Infants, and Children (WIC), were reported by the parent or guardian. Breastfeeding initiation, exclusivity through 6 months (only breast milk; no solids, water, or other liquids), and duration at 12 months were calculated among all infants and at the state level among black and white infants. Data were suppressed when the group’s sample size was <50 for that state. Breastfeeding estimates were weighted to adjust for multiple phone lines, mixed telephone use (landline and cellular), household nonresponse, and the exclusion of phoneless households, and accounted for the complex sampling design of NIS (*3*). Statistical analyses were conducted using chi-square tests to determine whether estimates for black infants were significantly different (p<0.05) from estimates for white infants.

Among all children born during 2010–2013, national estimates for breastfeeding initiation, exclusivity through 6 months, and duration at 12 months were 79.2%, 20.0%, and 27.8%, respectively (Table 1). Breastfeeding estimates varied by race/ethnicity, mother’s age and education, participation in WIC, and ratio of family income to the federal poverty threshold. Because black infants have consistently had the lowest rates of breastfeeding initiation and duration compared to other groups, the state-level estimates presented are limited to black and white infants (*2*).

**TABLE 1:** National prevalence of breastfeeding initiation, exclusive breastfeeding through age 6 months, and duration of breastfeeding at age 12 months* among children aged 19–35 months, by selected demographic characteristics — National Immunization Survey, United States, 2011–2015^†^

Characteristic	No. of respondents^§^	Initiated breastfeeding % (95% CI)	Breastfed exclusively through 6 months % (95% CI)	Breastfed at 12 months % (95% CI)
**Total**	88,436–90,692	79.2 (78.7–79.7)	20.0 (19.5–20.5)	27.8 (27.2–28.4)
**Child’s race/ethnicity**^¶^,**				
White, non-Hispanic	49,868–51,359	81.5 (80.9–82.1)	22.5 (21.9–23.1)	30.8 (30.1–31.5)
Black, non-Hispanic	9,091–9,255	64.3 (62.7–65.9)	14.0 (12.7–15.3)	17.1 (15.8–18.4)
Hispanic	17,775–18,075	81.9 (80.8–83.0)	18.2 (17.0–19.4)	26.3 (24.9–27.7)
**% of poverty level** ^††^				
<100	22,840–23,232	70.7 (69.6–71.8)	14.7 (13.8–15.6)	20.3 (19.3–21.3)
100–199	17,735–18,184	77.6 (76.5–78.7)	18.9 (17.9–19.9)	26.0 (24.8–27.2)
200–399	22,579–23,193	84.9 (84.1–85.7)	23.9 (22.9–24.9)	33.1 (32.0–34.2)
400–599	13,727–14,149	88.0 (87.1–88.9)	26.5 (25.1–27.9)	36.7 (35.2–38.2)
≥600	11,555–11,934	90.1 (89.2–91.0)	25.8 (24.1–27.5)	36.8 (35.0–38.6)
**Recipient of WIC**				
Yes	40,182–40,925	72.1 (71.3–72.9)	14.5 (13.8–15.2)	19.7 (18.9–20.5)
No (but eligible)	6,265–6,461	81.9 (79.9–83.9)	27.6 (25.6–29.6)	37.9 (35.7–40.1)
No (not eligible)	41,576–42,865	89.6 (89.1–90.1)	27.2 (26.4–28.0)	38.3 (37.4–39.2)
**Mother's education**				
Less than high school diploma or GED	9,329–9,496	68.8 (67.2–70.4)	14.5 (13.1–15.9)	21.8 (20.2–23.4)
High school diploma or GED	16,317–16,651	69.7 (68.5–70.9)	16.0 (15.0–17.0)	19.7 (18.6–20.8)
Some college	23,230–23,809	80.5 (79.6–81.4)	17.8 (16.8–18.8)	23.4 (22.3–24.5)
College graduate	39,560–40,736	91.1 (90.7–91.5)	27.7 (26.9–28.5)	40.3 (39.4–41.2)
**Mother's age (yrs)** ^¶^				
<20	760–768	60.1 (53.7–66.5)	7.3 (4.1–10.5)	8.7 (5.4–12.0)
20–29	32,148–32,841	74.0 (73.1–74.9)	16.4 (15.6–17.2)	19.8 (19.0–20.6)
≥30	55,528–57,083	83.5 (82.9–84.1)	23.1 (22.4–23.8)	34.3 (33.5–35.1)

**Abbreviations**: CI = confidence interval; GED = General Education Development certificate; WIC = Special Supplemental Nutrition Program for Women, Infants and Children.

* Breastfeeding initiation was determined based on response to the question, “Was [child] ever breastfed or fed breast milk?” Breastfeeding duration was assessed by asking, “How old was [child’s name] when [child’s name] completely stopped breastfeeding or being fed breast milk?” Exclusive breastfeeding was defined as only breast milk (no solids, no water, and no other liquids). To assess the duration of exclusive breastfeeding participants were asked two questions about age: 1) “How old was [child’s name] when they were first fed formula?” and 2) “How old was [child's name] when they were first fed anything other than breast milk or formula?” (including juice, cow's milk, sugar water, baby food, or anything else that [child] might have been given, even water). Breastfeeding duration and exclusivity rates are estimated among all infants included in the survey and not only infants whose mothers started breastfeeding.

^†^ Among children born during 2010–2013.

^§^ The number of respondents varies depending on the indicator.

^¶^ Differences in initiation, exclusive through 6 months, and at 12 months duration by demographic variables are statistically significant (p<0.05, chi-square test). Race/ethnicity test of significance was limited to non-Hispanic blacks and non-Hispanic whites.

** All racial/ethnic groups are included in the total in all other demographic breakdowns, but only the largest groups are presented here.

^††^ Ratio of self-reported family income to the federal threshold value, defined by the U.S. Census Bureau.

Among the 34 states* with sufficient sample size for analytic comparison, breastfeeding initiation ranged from 37.0% in Kentucky to 90.8% in Minnesota among black infants, and from 65.1% in Kentucky to 96.3% in DC among white infants. The state-specific percentage point differences (calculated as prevalence among white infants minus prevalence among black infants) in breastfeeding initiation between white and black infants ranged from −4.8 to 36.0, with substantial disparities in the South and Midwest. In 14 states, the difference in breastfeeding initiation between white and black infants was greater than 15 percentage points and the disparity exceeded 25 percentage points in seven of these states. The percentage point differences between white and black infants in exclusive breastfeeding through 6 months ranged from −4.2 in Rhode Island to 17.8 in Wisconsin, and at 12 months duration, the difference ranged from −4.4 in Minnesota to 31.6 in DC. A percentage point difference of ≥10 between white and black infants for 6 months of exclusive breastfeeding was observed in 12 states and for 12 months of breastfeeding in 22 states (Figure). These differences were significant (p<0.05) in each of these states (Table 2).

**FIGURE:**
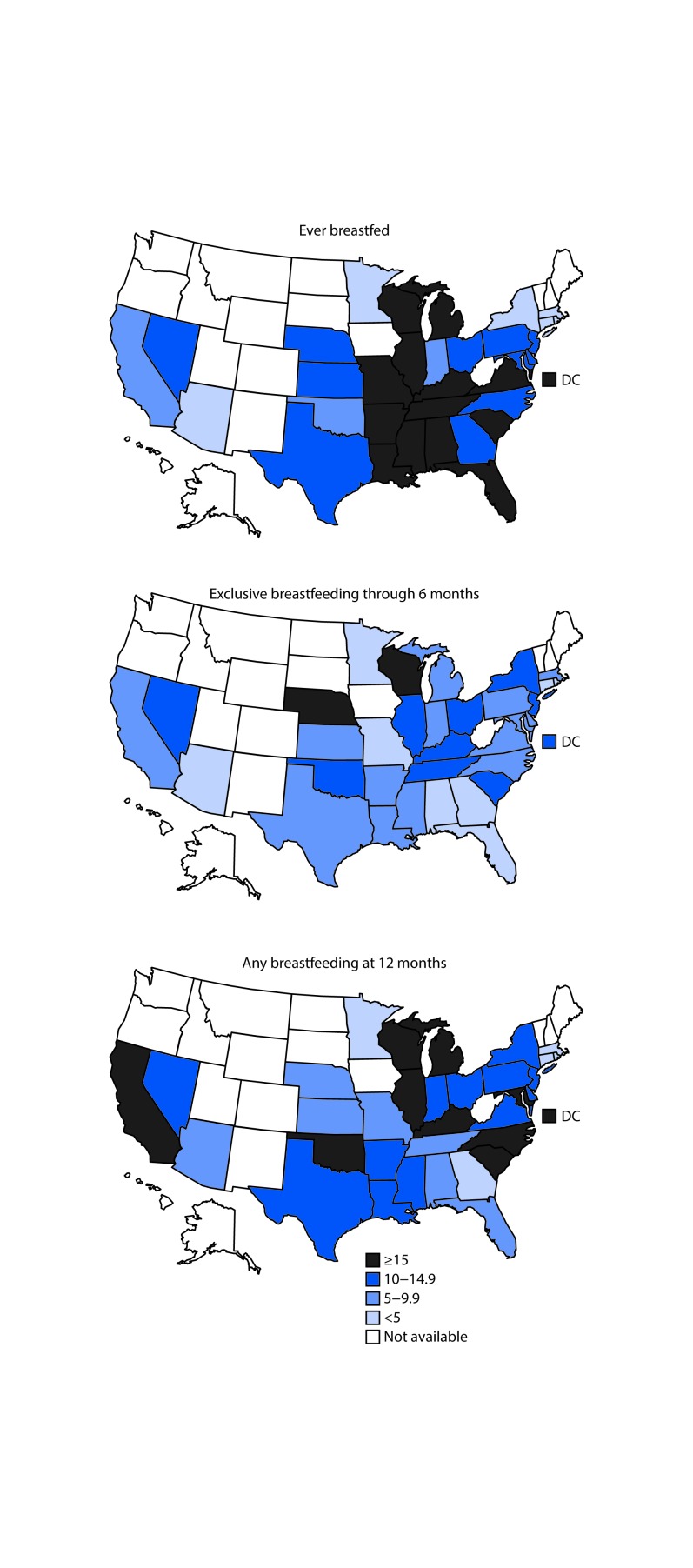
Percentage-point difference in breastfeeding indicators for non-Hispanic white and non-Hispanic black infants — National Immunization Survey, United States, 2011–2015*^,†^ * Among children born during 2010–2013. ^^†^^ Data were suppressed when the group’s sample size was <50 for that state.

**TABLE 2:** Prevalence of breastfeeding initiation, exclusive duration through age 6 months, and duration at age 12 months,* among children aged 19–35 months, by state and race — National Immunization Survey, United States, 2011–2015^†^

State	White, non-Hispanic				Black, non-Hispanic			
	No. of respondents^§^	Initiated breastfeeding % (95% CI)	Breastfed exclusively through 6 months % (95% CI)	Breastfed at 12 months % (95% CI)	No. of respondents^§^	Initiated breastfeeding % (95% CI)	Breastfed exclusively through 6 months % (95% CI)	Breastfed at 12 months % (95% CI)
Alabama	847–867	69.9 (65.8–74.0)	13.8 (11.2–16.4)	16.1(13.5–18.7)	305–308	52.5 (45.5–59.5)^¶^	12.2 (7.3–17.1)	11.0 (6.7–15.3)^¶^
Alaska	870–901	93.2 (91.2–95.2)	33.7 (30.0–37.4)	44.8 (41.0–48.6)	—**	—	—	—
Arizona	756–790	83.2 (79.5–86.9)	23.9 (20.0–27.8)	32.6 (28.5–36.7)	61–64	82.2 (70.1–94.3)	25.1 (10.4–39.8)	25.1 (11.1–39.1)
Arkansas	883–910	67.2 (63.0–71.4)	14.5 (11.6–17.4)	18.1 (15.2–21.0)	160–162	40.0 (30.5–49.5)^¶^	5.6 (1.9–9.3)^¶^	6.6 (2.7–10.5)^¶^
California	699–718	94.4 (91.7–97.1)	34.3 (28.9–39.7)	48.1 (42.4–53.8)	87–89	85.1 (75.6–94.6)	28.4 (14.3–42.5)	32.0 (17.9–46.1)^¶^
Colorado	1,020–1,056	90.2 (87.6–92.8)	28.3 (24.8–31.8)	40.0 (36.3–43.7)	—	—	—	—
Connecticut	870–912	85.0 (82.0–88.0)	23.1 (19.9–26.3)	28.3 (25.0–31.6)	131–139	81.6 (73.6–89.6)	19.9 (11.9–27.9)	27.9 (18.7–37.1)
Delaware	702–722	73.7 (69.8–77.6)	18.5 (15.3–21.7)	24.7 (21.2–28.2)	253–259	59.5 (52.3–66.7)^¶^	9.3 (5.6–13.0)^¶^	14.3 (9.8–18.8)^¶^
District of Columbia	677–700	96.3 (94.5–98.1)	27.7 (23.7–31.7)	47.9 (43.4–52.4)	569–582	65.5 (60.8–70.2)^¶^	14.8 (11.3–18.3)^¶^	16.3 (12.8–19.8) ^¶^
Florida	745–768	83.6 (80.0–87.2)	21.7 (17.7–25.7)	26.9 (22.8–31.0)	206–210	67.8 (59.9–75.7)^¶^	17.7 (11.1–24.3)	18.9 (12.1–25.7)^¶^
Georgia	669–685	74.8 (69.7–79.9)	16.7 (13.2–20.2)	20.1 (16.1–24.1)	394–404	61.1 (54.2–68.0)^¶^	17.2 (11.6–22.8)	17.0 (12.4–21.6)
Hawaii	282–285	92.4 (88.9–95.9)	37.5 (30.8–44.2)	55.1 (48.2–62.0)	—	—	—	—
Idaho	1,029–1,054	91.1 (89.0–93.2)	24.1 (21.1–27.1)	33.5 (30.2–36.8)	—	—	—	—
Illinois	1,653–1,691	81.4 (78.7–84.1)	20.6 (18.3–22.9)	28.1 (25.4–30.8)	536–544	58.7 (53.0–64.4)^¶^	9.7 (6.5–12.9)^¶^	12.6 (9.0–16.2)^¶^
Indiana	1,087–1,114	72.6 (69.3–75.9)	18.1 (15.3–20.9)	24.0 (21.1–26.9)	146–149	63.6 (53.9–73.3)	11.1 (5.1–17.1)^¶^	12.6 (6.8–18.4)^¶^
Iowa	1,053–1,084	79.7 (76.5–82.9)	21.4 (18.4–24.4)	26.6 (23.5–29.7)	—	—	—	—
Kansas	867–881	82.9 (79.9–85.9)	21.0 (17.9–24.1)	29.4 (26.0–32.8)	58	71.5 (57.5–85.5)	15.6 (2.5–28.7)	20.1 (6.6–33.6)
Kentucky	1,013–1,030	65.1 (61.3–68.9)	16.6 (13.9–19.3)	19.4 (16.6–22.2)	85	37.0 (24.3–49.7)^¶^	2.2 (0.2–4.2)^¶^	4.3 (0.6–8.0)^¶^
Louisiana	884–909	70.5 (66.8–74.2)	13.9 (11.3–16.5)	15.4 (12.7–18.1)	429–435	38.7 (33.2–44.2)	8.9 (5.8–12.0)^¶^	4.4 (2.5–6.3)^¶^
Maine	1,174–1,198	80.0 (77.0–83.0)	24.0 (21.2–26.8)	33.0 (29.9–36.1)	—	—	—	—
Maryland	878–907	85.4 (81.8–89.0)	28.2 (23.7–32.7)	37.6 (32.9–42.3)	494–506	75.4 (69.5–81.3)^¶^	19.0 (13.8–24.2)^¶^	21.4 (16.6–26.2)^¶^
Massachusetts	984–1023	84.4 (81.6–87.2)	23.7 (20.5–26.9)	35.2 (31.6–38.8)	75–79	89.2 (82.1–96.3)	18.5 (8.2–28.8)	35.8 (23.6–48.0)
Michigan	952–981	81.1 (77.8–84.4)	22.0 (18.7–25.3)	28.3 (24.9–31.7)	157–158	55.1 (45.1–65.1)^¶^	12.2 (6.1–18.3)^¶^	10.2 (5.2–15.2)^¶^
Minnesota	1,005–1,043	87.9 (85.2–90.6)	28.1 (24.7–31.5)	35.5 (31.9–39.1)	85–88	90.8 (83.6–98.0)	25.9 (14.4–37.4)	39.9 (25.9–53.9)
Mississippi	722–737	67.1 (62.6–71.6)	13.9 (10.6–17.2)	16.2 (12.9–19.5)	498–504	41.7 (36.1–47.3)^¶^	4.0 (2.1–5.9)^¶^	4.9 (2.6–7.2)^¶^
Missouri	983–1018	77.7 (74.4–81.0)	18.0 (15.2–20.8)	23.7 (20.7–26.7)	131–132	56.2 (45.9–66.5)^¶^	17.4 (9.7–25.1)	16.8 (9.1–24.5)
Montana	1,056–1,088	91.5 (89.3–93.7)	27.4 (24.0–30.8)	36.2 (32.6–39.8)	—	—	—	—
Nebraska	949–977	83.0 (79.9–86.1)	22.3 (19.3–25.3)	27.8 (24.6–31.0)	54–55	69.4 (55.0–83.8)	7.1 (1.1–13.1)^¶^	18.0 (7.1–28.9)
Nevada	703–724	83.7 (80.3–87.1)	24.1 (19.6–28.6)	29.8 (25.9–33.7)	105–110	69.9 (59.7–80.1)^¶^	11.8 (4.6–19.0)^¶^	19.0 (10.2–27.8)^¶^
New Hampshire	1,100–1,140	84.9 (82.4–87.4)	26.1 (23.1–29.1)	33.5 (30.4–36.6)	—	—	—	—
New Jersey	816–853	79.7 (76.0–83.4)	21.0 (17.4–24.6)	31.3 (27.4–35.2)	158–159	69.6 (60.9–78.3)^¶^	11.0 (5.3–16.7)^¶^	18.6 (11.6–25.6)^¶^
New Mexico	430–454	90.7 (87.2–94.2)	28.0 (22.7–33.3)	39.0 (33.5–44.5)	—	—	—	—
New York	1,632–1,695	83.3 (81.0–85.6)	22.6 (20.2–25.0)	36.9 (34.2–39.6)	412–423	80.8 (76.3–85.3)	11.9 (8.3–15.5)^¶^	23.6 (18.8–28.4)^¶^
North Carolina	865–892	80.0 (76.0–84.0)	22.2 (18.6–25.8)	32.4 (28.5–36.3)	274–277	66.0 (58.8–73.2)^¶^	16.2 (10.7–21.7)	17.4 (11.9–22.9)^¶^
North Dakota	1,273–1,308	84.3 (81.9–86.7)	22.3 (19.6–25.0)	24.9 (22.2–27.6)	—	—	—	—
Ohio	1,008–1,032	72.6 (69.0–76.2)	19.3 (16.4–22.2)	25.5 (22.3–28.7)	178–179	59.6 (51.0–68.2)^¶^	8.5 (3.8–13.2)^¶^	12.1 (6.6–17.6)^¶^
Oklahoma	756–782	76.6 (72.8–80.4)	19.4 (16.0–22.8)	25.7 (22.0–29.4)	105–106	70.3 (59.5–81.1)	7.4 (1.3–13.5)^¶^	9.0 (2.9–15.1)^¶^
Oregon	888–917	93.1 (91.1–95.1)	28.6 (25.2–32.0)	41.6 (37.9–45.3)	—	—	—	—
Pennsylvania	1,874–1,927	77.8 (74.9–80.7)	20.2 (17.6–22.8)	31.4 (28.4–34.4)	787–793	64.4 (59.0–69.8)^¶^	10.3 (7.7–12.9)^¶^	18.4 (13.7–23.1)^¶^
Rhode Island	826–856	77.0 (73.4–80.6)	20.3 (17.2–23.4)	27.9 (24.5–31.3)	74–77	79.8 (69.0–90.6)	24.5 (11.2–37.8)	25.8 (14.8–36.8)
South Carolina	871–885	75.2 (71.4–79.0)	21.1 (17.8–24.4)	25.0 (21.5–28.5)	319–321	55.1 (48.3–61.9)^¶^	10.0 (6.2–13.8)^¶^	8.1 (4.8–11.4)^¶^
South Dakota	922–938	85.4 (82.4–88.4)	23.4 (20.2–26.6)	28.8 (25.5–32.1)	—	—	—	—
Tennessee	969–992	73.8 (70.3–77.3)	17.4 (14.5–20.3)	20.0 (17.1–22.9)	180–188	55.5 (46.6–64.4)^¶^	7.3 (3.0–11.6)^¶^	14.4 (8.6–20.2)
Texas	2,065–2,121	83.8 (80.9–86.7)	23.6 (20.7–26.5)	30.2 (27.0–33.4)	560–568	70.1 (63.1–77.1)^¶^	16.3 (11.2–21.4)^¶^	17.6 (12.5–22.7)^¶^
Utah	1,063–1,119	91.5 (89.4–93.6)	22.1 (19.1–25.1)	39.5 (36.1–42.9)	—	—	—	—
Vermont	1,203–1,257	85.4 (83.0–87.8)	29.5 (26.7–32.3)	41.1 (38.1–44.1)	—	—	—	—
Virginia	1,153–1,178	84.2 (80.2–88.2)	23.7 (19.7–27.7)	36.5 (31.9–41.1)	217–221	67.0 (57.6–76.4)^¶^	14.8 (8.5–21.1)^¶^	22.4 (14.6–30.2)^¶^
Washington	856–886	94.2 (92.2–96.2)	28.0 (24.3–31.7)	39.2 (35.2–43.2)	—	—	—	—
West Virginia	1,230–1,246	61.1 (57.7–64.5)	11.8 (9.8–13.8)	16.0 (13.7–18.3)	—	—	—	—
Wisconsin	939–960	84.5 (81.5–87.5)	25.8 (22.5–29.1)	33.1 (29.6–36.6)	86–89	48.5 (34.9–62.1)^¶^	8.0 (2.2–13.8)^¶^	4.9 (0.8–9.0)^¶^
Wyoming	1,043–1,071	88.7 (86.1–91.3)	25.7 (22.5–28.9)	34.1 (30.7–37.5)	—	—	—	—

**Abbreviation**: CI = confidence interval.

* Breastfeeding initiation was determined based on response to the question, “Was [child] ever breastfed or fed breast milk?” Breastfeeding duration was assessed by asking, “How old was [child’s name] when [child’s name] completely stopped breastfeeding or being fed breast milk?” Exclusive breastfeeding was defined as only breast milk (no solids, no water, and no other liquids). To assess the duration of exclusive breastfeeding participants were asked two questions about age: 1) “How old was [child’s name] when they were first fed formula?” and 2) “How old was [child's name] when they were first fed anything other than breast milk or formula?” (including juice, cow's milk, sugar water, baby food, or anything else that [child] might have been given, even water). Breastfeeding duration and exclusivity rates are estimated among all infants included in the survey and not only infants whose mothers started breastfeeding.

^†^ Among children born during 2010–2013.

^§^ The number of respondents varies depending on the indicator.

^¶^ Referent group is non-Hispanic whites. Estimate is statistically significant (p<0.05).

** Not available. Data were suppressed when sample size was <50.

## Discussion

National estimates of breastfeeding initiation and duration have consistently improved among black and white infants over the past decade (*2*); however, the difference in breastfeeding rates between black and white infants remains substantial. Among infants born during 2010–2013, the gap in breastfeeding initiation between black and white infants was 17.2 percentage points, only slightly less than the 19.9 percentage point difference between black and white infants born during 2003–2006 (a timeframe when the methodology only included the landline sample) (*4*). The percentage point difference in the rate of exclusive breastfeeding through 6 months between black and white infants was 7.8 for children born during 2003–2006 (CDC, Nutrition Branch, unpublished data, 2016), and 8.5 for infants born during 2010–2013. The percentage point difference in the rate of breastfeeding at 12 months between black and white infants was 9.7 among infants born during 2003–2006 and 13.7 among infants born during 2010–2013 (*4*).

Multiple factors influence a woman’s decision to start and continue breastfeeding. Lack of knowledge about breastfeeding, unsupportive cultural and social norms, concerns about milk supply, poor family and social support, and unsupportive work and childcare environments make it difficult for many mothers to meet their breastfeeding goals (*5*). Certain barriers are disproportionately experienced by black women (e.g., earlier return to work, inadequate receipt of breastfeeding information from providers, and lack of access to professional breastfeeding support), (*6*). For example, although evidence-based maternity care practices that support breastfeeding have been reported to increase breastfeeding initiation, exclusivity, and duration (*5*), black mothers might not have consistent access to these supportive practices. A study of hospital support for breastfeeding indicated that facilities located in zip codes with higher percentages of black residents than the national average were less likely to meet five indicators for supportive breastfeeding practices (early initiation of breastfeeding, limited use of breastfeeding supplements, rooming-in, limited use of pacifiers, and post-discharge support), than those located in areas with lower percentages of black residents (*7*).

In 2011, *The Surgeon General’s Call to Action to Support Breastfeeding* outlined 20 action steps to support breastfeeding across various sectors of society, including a call to better understand and address breastfeeding disparities (*5*). A U.S.-based review of randomized trials evaluating breastfeeding interventions targeting minorities showed that group prenatal education, peer counseling interventions, breastfeeding-specific clinic appointments, and enhanced hospital practices/WIC-based services positively affected breastfeeding outcomes among minority women (*8*). CDC is currently funding a hospital-based quality improvement initiative designed to support hospitals to implement evidence-based maternity care practices. Currently 93 U.S. hospitals participate in EMPower Breastfeeding: Enhancing Maternity Practices^†^ in 24 states, primarily in the South and Midwest, where the disparities in breastfeeding rates between black and white infants is greatest.

The findings in this report are subject to at least three limitations. First, estimates do not account for other factors potentially associated with lower breastfeeding rates among black infants, e.g. in-hospital formula feeding and socioeconomic characteristics such as percentage of poverty level and participation in WIC. However, previous analyses have indicated that racial differences exist that are independent of socioeconomic and demographic factors (*9*). Nevertheless, because the racial disparity in breastfeeding might depend on factors such as income and education, future studies examining the interactions among these factors are warranted to understand the independent contribution of each factor. Second, breastfeeding behaviors were self-reported by the respondent retrospectively when the child was aged 19–35 months, which could be subject to recall bias and social desirability. However, maternal recall for estimating breastfeeding initiation and duration is a reasonably valid and reliable method (*10*). Finally, despite combining survey years, in 17 states, the sample size for black infants was less than 50, limiting the ability to assess racial differences in all states.

The difference in breastfeeding indicators among black and white infants by state continues to be substantial. Though certain interventions targeting black families have positively affected breastfeeding outcomes, additional research is needed to better understand the underlying factors contributing to the widespread persistence of the gap in breastfeeding rates by race (*6*,*8*). To reduce the disparities in rates of breastfeeding between black and white infants, interventions need to specifically address breastfeeding barriers experienced disproportionally by black mothers (*6*).

**Summary**

**What is already known about this topic?**

The American Academy of Pediatrics recommends exclusive breastfeeding for the first 6 months of a baby’s life and continued breastfeeding with complementary foods until age ≥12 months. Over the past decade, national estimates of breastfeeding initiation and duration have consistently improved among both non-Hispanic black (black) and non-Hispanic white (white) infants; however, differences in breastfeeding rates by race have persisted.

**What is added by this report?**

Differences in breastfeeding rates between black and white infants vary by state, and rates are lower among blacks in most states. Breastfeeding initiation rates were significantly lower among black infants in 23 states; in 14 of these states, the difference was at least 15 percentage points. A significant difference of at least 10 percentage points in exclusive breastfeeding through 6 months was found between black and white infants in 12 states, and at 12 months of breastfeeding in 22 states.

**What are the implications for public health practice?**

To increase the rate of breastfeeding among black infants, interventions are needed to address barriers experienced disproportionately by black mothers, including earlier return to work, inadequate receipt of breastfeeding information from providers, and lack of access to professional breastfeeding support. Enhanced understanding of these barriers could improve the effectiveness of interventions.

## Footnotes

* Alabama, Arizona, Arkansas, California, Connecticut, Delaware, District of Columbia, Florida, Georgia, Illinois, Indiana, Kansas, Kentucky, Louisiana, Maryland, Massachusetts, Michigan, Minnesota, Mississippi, Missouri, Nebraska, Nevada, New Jersey, New York, North Carolina, Ohio, Oklahoma, Pennsylvania, Rhode Island, South Carolina, Tennessee, Texas, Virginia, and Wisconsin.

† http://empowerbreastfeeding.org.
