# *Erratum:* Vol. 66, No. 27

**DOI:** 10.15585/mmwr.mm6630a5

**Published:** 2017-08-04

In “Racial and Geographic Differences in Breastfeeding—United States, 2011–2015,” on page 723, in the first paragraph, the fifth sentence should have read, “Among the 34 states (including the District of Columbia [DC]) with sufficient sample size (≥50 per group), initiation rates were significantly (p<0.05) lower among black infants than white infants in **22** states; in 14 of these states (primarily in the South and Midwest), the difference was at least 15 percentage points.”

On page 727, under “What is added by this report?” the second sentence should read, “Breastfeeding initiation rates were significantly lower among black infants in **22** states; in 14 of these states, the difference was at least 15 percentage points.”

